# Comparing social stigma of anorexia nervosa, bulimia nervosa, and binge-eating disorder: A quantitative experimental study

**DOI:** 10.1186/s40337-025-01198-x

**Published:** 2025-01-27

**Authors:** Carlye S. Aird, Bennett A. A. Reisinger, Stephanie N. Webb, David H. Gleaves

**Affiliations:** 1 University of South Australia, Justice & Society, Adelaide, South Australia Australia; 2 University of South Australia, St Bernards Rd, Room C1-07 Magill Campus, Magill, SA 5072 Australia

**Keywords:** Eating disorder, Anorexia nervosa, Bulimia nervosa, Binge-eating disorder, Stigma

## Abstract

**Background:**

Currently, we know little regarding how stigma attributed to eating disorders compares to that of other psychological disorders and additionally within different types of eating disorders. In the current study, we aimed to explore the stigmatisation of eating disorders by comparing the stigma attributed to anorexia nervosa, bulimia nervosa, and binge-eating disorder, utilising depression as a comparative control.

**Methods:**

A total of 235 participants from the general population were randomly assigned to an anorexia nervosa, bulimia nervosa, binge-eating disorder, or depression condition. Participants responded to a questionnaire consisting of several adapted versions of pre-existing subscales that measured levels of stigma associated with psychological disorders generally, as well as stigma associated with eating disorders specifically. We used several one-way analyses of variance to investigate the differences in stigma attributed towards the aforementioned psychological disorders.

**Results:**

Results suggested that all three eating disorders were significantly more stigmatised than was depression. Between the eating disorders, the three were generally equivalent except that binge-eating disorder was significantly more stigmatised than both anorexia nervosa and bulimia nervosa on a subscale measuring trivialness.

**Conclusions:**

These findings indicate that individuals with eating disorders, including binge-eating disorder, may be at a higher risk of experiencing the negative implications of stigma when compared to other psychological disorders, such as depression. To our knowledge, this study is one of few that directly quantify and compare stigma attributed towards anorexia nervosa, bulimia nervosa, and binge-eating disorder. Through further research, a better understanding around the expression of stigma towards specific eating disorders could inform the development of targeted interventions to help reduce the stigma associated with these disorders. This knowledge could also advance the understanding of the lived experience of individuals living with eating disorders, subsequently informing treatment practices.

## Abstract (plain language summary)

We conducted this study to better understand how people view different mental health conditions, specifically depression and the eating disorders anorexia nervosa, bulimia nervosa, and binge-eating disorder. We collected eligible responses from 235 people from the general public regarding their attitudes towards these disorders. We found that all three eating disorders were significantly more stigmatised than depression. We also found that binge-eating disorder was slightly more stigmatised than anorexia nervosa and bulimia nervosa in certain areas. These results suggest that people living with eating disorders, including binge-eating disorder, may face higher levels of stigma compared to those living with other mental health conditions, like depression. These findings are important as they can help us understand how society views different mental health conditions. In doing so, we can work towards implementing effective initiatives aimed at reducing stigma and improving support for people living with eating disorders.

## Background

Psychological disorders have long been associated with stigma, resulting in the marginalisation and social oppression of those affected [1]. Consequently, individuals diagnosed with psychological disorders face challenges associated with the disorder itself and additional implications of negative stereotypes, prejudice, and discrimination [2]. These implications are often costly to the individual and can further exacerbate the symptoms of the psychological disorder through experiences of isolation, self-stigma (the internalisation of negative public attitudes), lowered self-esteem, lowered self-efficacy, hesitancy to seek professional support, and poor treatment adherence [2, 3, 4]. Consequences of stigma highlight the necessity of understanding the experience of stigma attributed towards psychological disorders, particularly in the case of disorders where stigma research is lacking.

Stigma involves any negative evaluation or differentiation owing to a particular condition, group membership, or state of life [1]. Although multiple forms of stigma exist (see, Grappone [5] for a review); the current study focused on social stigma, which is when a person holds negative prejudices and stereotypes against individuals resulting in intolerance and discrimination [3]. Power imbalances feed the social stigmatisation process, whereby society devalues and disapproves of stigmatised individuals due to an attribute that does not align with what society considers the norm [1]. Thus, the manifestation of social inequality further marginalises minority groups and assists with the maintenance of stigma. In the context of psychological disorders, stigma may present as believing that every individual with a psychological disorder is weak or unpredictable, having unfavourable attitudes such as anger or pity towards individuals with psychological disorders, or the active avoidance, exclusion, or intolerance of individuals with psychological disorders.

A variety of factors influence stigma towards psychological disorders. Allport [6] denoted that a lack of contact and knowledge is critical in both generating and maintaining stigma. Penn and Couture [7] found that mental health knowledge was negatively correlated with stigma towards individuals with psychological disorders. Additionally, myths and misinformation further reinforce higher levels of social stigma, particularly in instances where individuals have minimal real-life exposure to psychological disorders or other mental health conditions [6]. Media such as films and news reports commonly perpetuate such misinformation by inaccurately depicting psychological disorders and their symptoms [8].

Knowledge of the perpetuation of stigma towards psychological disorders is currently well established. However, how this stigma differs between different psychological disorders is less understood. Reisinger and Gleaves [9] found that stigma levels varied across psychological disorders, with certain disorders being associated with significantly higher levels of social stigma. Current literature suggests that eating disorders (EDs) are among such disorders that are highly stigmatised; however, the comparative levels of stigma attributed to the different presentations of EDs remains under-researched [2, 10].

## Stigma and eating disorders

Eating disorders are characterised by a persistent disturbance in eating or eating-related behaviours that result in significant impairment of physical health and/or psychosocial functioning [11]. As of 2023, 4.45% of the Australian population, equating to over 1.1 million individuals, were living with an ED [12]. Furthermore, ED prevalence is growing at an alarming rate, especially among young Australians, as demonstrated by a 62% increase in ED presentations among children and adolescents between 2018 and 2020 [13]. Three main EDs are anorexia nervosa (AN), bulimia nervosa (BN), and binge-eating disorder (BED), with the National Eating Disorder Collaboration estimating that the Australian prevalence of these EDs is 0.16%, 0.51%, and 0.94% respectively [12]. AN has the highest mortality rate of all psychological disorders due to its associated physiological effects and increased suicidality [14]. Given the increasing prevalence and associated high mortality rates, investigating the relationship between EDs and stigma is crucial.

In exploring stigma towards individuals with EDs in general, regardless of ED type, researchers have identified several central themes. People often attribute personal responsibility for the ED directly to the individual with the disorder [2, 4, 10, 15]. Additionally, themes of attention-seeking, weakness of character, and it being a disorder that individuals can easily overcome, are all prominent attitudes associated with EDs [2]. The belief that having AN or BN “might not be too bad” is also common, with some individuals even showing admiration for certain aspects of EDs ([16], p. 520; [17], p. 88). Roehrig and McLean [10] demonstrated such beliefs exist when they found that over half of their participants indicated that it “might not be too bad” (p. 673) to be like the individual with an ED illustrated in a vignette. Additionally, the majority of participants reported that they admired certain aspects of the illustrated ED, such as the rigidity in controlling eating and exercising every day, whereas some reported that they could be motivated to imitate ED behaviours in their own lives. These findings reflect complex stigmatising attitudes, where EDs are not considered to be debilitating psychological disorders, but instead, their symptoms are minimised, and in some cases revered. For individuals with lived experience of EDs, such beliefs can lead to significant psychological, social, physical, and behavioural consequences, including social isolation, avoidance of treatment, and exacerbation of symptoms [2].

Current literature alludes to differences in the stigmatisation of EDs according to the type of ED. Across several different studies, AN appears to elicit higher expressions of negative attitudes such as irritation, anger, lack of sympathy, and feelings of uncomfortableness during interactions, whereas feelings of sympathy and more supportive attitudes have been associated with BN [2, 16]. Findings from Roehrig and McLean [10] suggested that, among a sample of psychology students, AN elicited greater levels of stigma than did BN. Roehrig and McLean [10] proposed that the visibility of AN symptoms, namely emaciation, may have contributed to the higher stigmatisation of AN compared to BN. Although untested, such reasoning would suggest that BED would be attributed with lower levels of stigma compared to AN; however, stigma associated with obesity may actually have the opposite effect [18]. Hollett and Carter [18] highlighted the complicating role of weight stigma, showing that obesity may exacerbate the stigma associated with BED. Although some disordered eating behaviours, such as strict dietary control, may be admired, higher body weight can be associated with negative stereotypes like “laziness” or “carelessness”. These stereotypes may contribute to a unique form of stigma that differs from the stigma associated with AN and BN [18, 19].

Comparatively, the stereotype in which an individual with an ED is believed to be responsible or blamed for their disorder is most prevalent towards BN [2]. Using a sample of university students from the United States, Wingfield et al. [20] found that the participants perceived individuals with BN to be more self-destructive and responsible for their condition, whereas individuals with AN were perceived to have greater self-control. These findings do however vary within the literature. A lack of direct comparisons between EDs limits our ability to adequately explain contradicting findings, thus making it difficult to postulate which EDs may be associated with higher levels of stigma.

Further contradictions are evident when considering the stigma associated with BED in comparison to AN and BN. For example, in a scoping review, Brelet et al. [2] found that individuals with BED experienced equal or more negative attitudes than both AN and BN, whereas other researchers have contrastingly found that AN and BN are associated with more negative attitudes [21]. Previous research implies that lower levels of stigma associated with BED may be a function of individuals with BED being perceived as less functionally impaired than those with AN or BN [21]. Additionally, both AN and BN are more highly associated with social distancing, avoidance behaviours, and reluctance in job interviewing, this being particularly relevant to AN [2, 16]. In comparison to AN and BN, the establishment of BED as an independent diagnosable psychological disorder in the *DSM* is somewhat recent [22]. As a result, research surrounding BED is still in its infancy, making inconsistencies in the relevant stigma literature unsurprising.

The degree of stigma attributed to EDs relative to other psychological disorders is also largely unknown, particularly in relation to BED. Roehrig and McLean [10] found that both AN and BN appeared to attract more stigmatising attitudes compared to depression.^1^ Roehrig and McLean [10] found that, when compared to depression, people reported individuals with EDs, particularly AN, to be more responsible for and in control of their symptoms, more fragile, and more likely to be using their disorder as a mechanism to garner attention. Additionally, Stewart et al. [23] noted that people reported experiencing greater discomfort when interacting with an individual with AN in comparison to an individual with depression. Although stigma still exists towards depression, it appears to be lesser than that attributed to EDs. Previous findings have similarly indicated that stigma levels associated with depression are relatively lower compared to other psychological disorders [9, 10, 23, 24].

## The current study

Current ED stigma research primarily focuses on AN and BN, yet comparisons between these two EDs are limited, as is the consideration of BED. Considering the drastic implications of ED stigma in combination with the disorders’ high mortality rate and/or health complications, there is a drastic need for further research that directly explores and compares stigma associated with AN, BN, and BED. As depression appears to be the most widely used control disorder within stigma research [10], the current study will include depression as a control disorder to indicate the relative levels of stigma associated with AN, BN, and BED. Exploring the differences in stigma levels associated with EDs and how these compare to a well-established disorder in stigma research, like depression, allows for a more comprehensive understanding of ED stigma.

The aim of the current study was to compare the levels of stigma associated with AN, BN, and BED, using depression as a comparison. We hypothesised that AN, BN, and BED would be attributed with higher levels of stigma than depression. The following research question was also proposed; what, if any, are the differences in levels of stigma associated with AN, BN, and BED respectively, and how do these compare to the level of stigma associated with depression?

## Methods

### Participants

To be eligible to participate in this study, participants had to be 18 years or older and living in Australia at the time of data collection. The initial sample comprised 343 responses. From this, the data of 235 participants were used. Participants responded to flyers displayed throughout two university campuses and posted on social media. Snowballing methods were also utilised to increase the sample size. Of the included sample, the majority of participants reported being female (83.83%), university educated (68%), and identified as Anglo-Australian (61%). Participants’ ages ranged from 18 to 66 with a mean of 29.56.

### Materials

Prior to the current research, the featured scales utilised several different response formats. To maintain consistency and minimise participant burden, for the current study, we altered these scales so that all items utilised a semantic differentiated forced-response scale (1 = strongly disagree, 2 = disagree, 3 = slightly disagree, 4 = slightly agree, 5 = agree, 6 = strongly agree). As such, higher scores on the scales indicated higher levels of stigma. The original authors of all scales granted permission for the adaptation and use of their scales in this study. Items remained fundamentally the same, except where the original scales referred to “mental illness” more generally, the adapted versions referred to AN, BN, BED, or depression respectively. For example, where an original scale item would read “I would find it hard to talk to someone who has a mental illness”, the adapted scale item for the BN condition would read “I would find it hard to talk to someone who has bulimia nervosa”. Similar adaptions to each of these scales have been used previously to assess stigma attributed to other specific psychological disorders [26, 27, 28, 29, 30].

#### Demographics

As part of the survey, participants indicated their age (in years), gender (male, female, or non-binary), country of residence, ethnicity, and level of education (university educated or not university educated).

#### Stigma towards psychological disorders

To measure stigma attributed towards the three EDs and depression, adapted versions of the Stigma and Self-Stigma Scale (SASS; [31]) and the Opinions Scale (OS; [32]) were utilised to create a combined composite stigma score through calculating a total mean score of combined items. Cronbach’s alpha scores for this adapted scale, based on the current data, are in Table 1.

The SASS measures aspects of stigmatising ideations towards psychological disorders by having participants indicate their level of agreement with various statements. The original SASS consists of 42 items with six subscales. However, because not all of these subscales are relevant to ED or depression stigma, we only used two subscales, comprising 12 total items. These subscales were the Stigma to Others subscale (e.g., “People with mental disorders are not really ill”), and the Anticipated Stigma subscale (e.g., “If I had a mental disorder, I would worry other people would think I was weak”). Docksey et al. [31] demonstrated acceptable internal consistency scores for the Stigma to Others (*α* = .71) and Anticipated Stigma (*α* = .88) subscales in a sample of 328 UK government employees.

The original OS is a 7-item scale that measures attitudes towards individuals with a psychological disorder [32]. Due to two original items appearing to be irrelevant to EDs or depression (the “danger to others” and “unpredictable” items), we removed them, but we added an additional item, (“[the target] is acting the way they do for attention”), as Stewart et al. [33] did when they measured and compared perceptions towards healthy individuals and individuals with AN, schizophrenia, and asthma. This resulted in a new adapted OS comprising five original items from Crisp et al. [32] and one new item from Stewart et al. [33].

#### The eating disorder stigma scale (EDSS; [34])

To measure stigma attributed specifically towards EDs, we used the EDSS. Cronbach’s alpha scores for this scale, based on the current data, are in Table 1.

Crisafulli et al. [34] primarily developed the EDSS to assess a variety of beliefs individuals may hold towards AN. As such, the EDSS encapsulates unique elements of ED stigma which are often omitted from more general psychological disorder stigma scales (e.g., vanity). The scale comprises 20 items which fall into four subscales: trivial (e.g., “anorexia nervosa is not as serious as other mental illnesses”), selfish/vain (e.g., “a person with anorexia nervosa is selfish”), weak (e.g., “a person with anorexia nervosa is weak”), and blame (e.g., “a person with anorexia nervosa caused their own eating disorder”). Within university student samples, previous research has indicated acceptable internal consistencies for the entire scale (*α* = .95) and the individual subscales (between *α* = .80 −.92) [26, 27, 34]. We excluded the blame subscale from the current study due to unacceptable internal consistency with the current sample (see Table 1).

### Design

To address the proposed research question and hypothesis, we used a between-groups experimental design, with participants randomly assigned to one of four conditions, each based on one of the four different variations of a questionnaire which assessed stigma towards psychological disorders (AN, BN, BED, or depression respectively).

### Procedure

The researchers received ethics approval from the University of South Australia’s Human Research Ethics Committee (protocol number 205469) prior to data collection. Initially, participants read information about the study via the Qualtrics survey platform. Subsequently, all willing participants provided electronic informed consent before they proceeded to the survey. Participants answered a series of demographic questions and were then randomly assigned to one of the four conditions. Participants then read a brief definition of their allocated disorder (AN, BN, BED, or depression [control group]) and then answered the corresponding adapted survey. Participants who were part of the AN, BN, or BED conditions answered additional questions to the depression condition whereby they responded to an additional scale focusing on ED stigma. Participants who completed the survey had the option to enter a draw to win one of five $60 gift vouchers.

### Data analysis

#### Missing and unusable data

The initial sample comprised 343 responses. Of these, we removed 108 responses because they were incomplete (i.e., participant attrition) or manually identified as fraudulent bot responses (i.e., computer programs that complete online surveys). We used bot detection measures, such as CAPTCHA and specific detection items, to identify bot responses. One example of this was cross-referencing the provided postcode with the provided state of residence. We used a forced response system, preventing participants from skipping items. Consequently, any missing data were typically due to participant dropout. The final sample comprised *N* = 235 participants.

#### Data entry and randomisation check

The data were entered into version 29 of IBM SPSS Statistics and all tests of the relevant assumptions of all analyses were conducted, after which the statistical tests proceeded accordingly. We conducted a randomisation check, using age and gender, to ensure participant randomisation between the four conditions. The distributions of gender and age were equivalent, thereby indicating the randomisation process was adequate. Scores were on a scale ranging from −3 to 3, for ease of stigma identification, where −3 indicated lowest stigma and 3 indicated highest stigma.

#### Analyses conducted

To determine differences in stigma attributed towards AN, BN, BED, and depression, we conducted several one-way analyses of variance (ANOVAs) on a SASS and OS composite total stigma score and subscale scores. To assess differences in stigma attributed towards AN, BN, and BED more specifically, we conducted ANOVAs on EDSS total and subscale scores. Where ANOVAs were statistically significant (*p* <.05), we used Tukey’s post-hoc tests to determine which groups differed from which.

## Results

### Demographics

The majority of participants reported being female (197, 83.83%; 33, 14.04% male and five non-binary), were university-educated (*n* = 160, 68.1%), and identified as Anglo-Australian (*n* = 143, 60.9%). Fifty-four (23%; mean age 29.56 years, *SD* = 12.09) participants were assigned to the AN condition, 61 (26%; mean age 27.46 years, *SD* = 11.13) to the BN condition, 59 (25.1%; mean age 29.07 years, *SD* = 9.81) to the BED condition, and 61 (26%; mean age 32.15 years, *SD* = 13.86) were assigned to the depression condition.

### Score reliability analysis

Score reliability analyses were conducted to assess the internal consistency of the modified scales and subscales within the utilised sample. Cronbach’s alpha scores, based on the current data, are in Table 1.

**Table 1:** Internal consistency estimates for the SASS/OS Composite, EDSS Total, and EDSS Subscale scores

Scales	Total α	Condition’s α			
		AN	BN	BED	Depression
SASS/OS (Total)	.86	.84	.87	.86	.85
EDSS Total	.94	.92	.96	.94	N/A
EDSS Trivial	.94	.92	.97	.93	N/A
EDSS Selfish/Vain	.90	.89	.92	.90	N/A
EDSS Weak	.93	.91	.95	.93	N/A
EDSS Blame	.56	.42	.67	.51	N/A

Note. N/A denotes that scale was not administered. AN = anorexia nervosa; BN = bulimia nervosa; BED = binge-eating disorder; SASS = Stigma and Self-Stigma Scale; OS = Opinions Scale; EDSS = Eating Disorder Stigma Scale.

### Main analysis

#### Comparing stigma between eating disorders and depression

To assess differences in the stigmatisation of EDs and depression, we conducted a one-way ANOVA with a composite SASS and OS stigma score as the dependent variable. Means and standard deviations of the composite SASS and OS scores of the AN, BN, BED, and depression conditions are in Table 2. There was a significant main effect of condition, *F*(3,231) = 3.71, *p* = .012, *η*^2^ = .046. Tukey post-hoc tests suggested that stigma scores in the depression condition were significantly lower than those of the AN, BN, and BED conditions.

**Table 2:** Comparisons of the SASS/OS Composite, EDSS Total, and EDSS Subscale score means of the AN, BN, BED, and Depression experimental conditions

Scales	Condition				*F*	*η* ^2^
	AN *n* = 54	BN *n* = 61	BED *n* = 59	Depression *n* = 61		
SASS/OS Composite						
Mean	-0.89^a^	-0.92^a^	-0.90^a^	-1.28^b^	3.71^**^	.05
Std. Dev.	0.76	0.78	0.77	0.71		
EDSS Total						
Mean	-2.21^a^	-2.09^a^	-1.88^a^	N/A	1.64	.02
Std. Dev.	0.93	1.39	0.96			
EDSS Trivial						
Mean	-2.30^a^	-2.12^a^	-1.57^b^	N/A	6.59^*^	.07
Std. Dev.	0.95	1.17	1.23			
EDSS Selfish/Vain						
Mean	-2.11^a^	-2.04^a^	-1.98^a^	N/A	0.24	<.01
Std. Dev.	1.00	1.06	0.98			
EDSS Weak						
Mean	-2.24^a^	-2.12^a^	-2.09^a^	N/A	0.27^*^	<.01
Std. Dev.	1.11	1.29	1.09			

Note. Numerically higher means represent greater levels of stigma. N/A denotes that scale was not administered. AN = anorexia nervosa; BN = bulimia nervosa; BED = binge-eating disorder; SASS = Stigma and Self-Stigma Scale; OS = Opinions Scale; EDSS = Eating Disorder Stigma Scale

**p <.001. *p <.05.

Means with same superscript are not statistically significantly different at *p* <.05 level

#### Stigma specifically towards eating disorders

To assess any differences in stigma attributed towards the three EDs AN, BN, and BED more specifically, we conducted a one-way ANOVA to examine the effect of condition on participants’ total EDSS scores. Means and standard deviations of EDSS scores for the AN, BN, and BED conditions are in Table 2. The main effect of condition was not statistically significant, *F*(2,171) = 1.64, *p* = .197, *η*^2^ = .019, and Cohen’s *d* values ranged from 0.10 to 0.35.

In order to determine whether there were any differences within the EDSS subscales between the three ED conditions, we conducted three additional one-way ANOVAs (see also Table 2). There was a significant main effect of condition on the Trivial subscale, *F*(2,171) = 6.59, *p* = .002, *η*^2^ = .072; however there was no significant effect of condition on the Selfish/Vain, *F*(2,171) = 0.24, *p* = .786, *η*^2^ = .003 or Weak subscales, *F*(2,171) = 0.27, *p* = .762, *η*^2^ = .003. Tukey post-hoc test results on the Trivial subscale of the EDSS indicated a significant difference between the AN and BN conditions and the BED condition, with significantly higher levels of stigma being attributed towards BED compared to both AN and BN on this subscale.

## Discussion

The purpose of this study was to explore the stigma levels associated with EDs, and how these compared to the stigma levels of other psychological disorders, in this case depression. As predicted, all three EDs were associated with significantly more stigma than was depression, with medium effect sizes. However, the differences between the EDs, on all but one of the variables, were small and not statistically significant.

The finding that EDs had higher levels of stigma than did depression is consistent with previous research (e.g [10, 23]). Although researchers commonly use depression within stigma research to provide a relative standing to which other psychological disorders are stigmatised, this is not to say that individuals with depression do not experience stigma [9, 23, 24]. The current research instead suggests EDs may be stigmatised at a higher level than is depression. One reason for this finding could be the increased exposure to and development of knowledge about depression as a psychological disorder within the general population [35]. Although existing research supports these findings, few studies have empirically compared the stigma attributed to EDs with other psychological disorders, thus we attempted to provide a preliminary indication of the relative standing of ED stigma.

Based on group comparisons, participants reported significantly higher levels of stigma towards all three EDs compared to depression; however, stigma levels among the three EDs did not differ significantly. Although not statistically significant, the SASS and OS composite stigma scores indicated AN was the most stigmatised ED in comparison to depression, which was consistent with previous findings [10]. The visible nature of AN symptoms, such as emaciation, could explain why AN is more stigmatised than depression. Previous research suggests that differences in acts of behavioural discrimination, such as discomfort and avoidance in interpersonal interactions, demonstrates the stigma differences evident between AN and depression [2, 23]. Brelet et al. [2] and Stewart et al. [23] proposed that insecurities about a person’s self-image may arise when interacting with someone of low weight or serious body shape issues. For example, an individual may compare their own body weight or shape with someone who is underweight and feel comparatively larger, resulting in feelings of inadequacy and jealousy. Alternatively, an individual may develop feelings of uncomfortableness or disgust when interacting with someone who is emaciated, due to a lack of understanding around the disorder [2]. Additionally, the perceived high emphasis on vanity associated with AN and BN could also offer an explanation as to the differences in stigma levels from depression [2]. A similar rationale may explain why BED may be attributed higher levels of stigma than is depression, in that physical and visible symptoms such as obesity are often associated with BED [2, 36, 37].

It is also plausible to suggest that, as knowledge and information around depression continues to grow, the level of stigma associated with depression will continue to be relatively lower than other lesser-understood psychological disorders such as EDs. Similarly, higher prevalence rates of depression could suggest that general exposure to depression within society is higher than that of EDs, resulting in lower levels of stigma [35]. As such, further stigma research could be useful in exploring the effect of increased exposure to ED information and facilitating increased awareness and understanding around EDs.

Despite EDs being significantly more stigmatised than depression, there were no statistically significant differences between the EDs, when using the SASS and OS. However, when investigating the subtleties of these disorders through the subscales of the EDSS, we found that, in terms of trivialness, BED was significantly more stigmatised than both AN and BN. Higher scores on the Trivial subscale suggest an attitude that the disorder is less serious, less important, or less impactful compared to other psychological disorders. As such, it is possible that the severity of BED is dismissed to a greater extent than evident in AN and BN.

A contributing reason behind the associated triviality could be the novelty of BED as a distinct disorder in the *DSM.* That is, there may not have been enough time to increase community understanding and knowledge of it as a mental illness (e.g., as opposed to the simple stereotype that someone is ‘lazy’). It is only from the *DSM-5* [22] onwards that BED received full diagnostic status as its own category of ED. Prior to this, in the *DSM-IV* [38], BED was not an independent diagnosable mental disorder, but rather was in Appendix B: Criteria Sets and Axes Provided for Further Study. It is also of note that the inclusion of BED as a distinct psychological disorder has been criticised and the topic of some debate. Such criticisms include its distinguishableness from obesity; the stringency of the binge-eating frequency and duration criterion; the role of overevaluation of weight/shape; the degree to which comorbidities, other symptoms, and functional disabilities influence disorder severity; and the lack of clarity regarding subjective binge-eating [37]. Although empirical evidence appears to mostly support the recognition and diagnosis of BED, future research remains imperative to resolve such challenges. The effects of only recently being included in the *DSM* also include historically lesser research interest compared to AN and BN, thereby resulting in lesser public awareness and understanding [2]. In this context, higher levels of associated trivialness may demonstrate a perceived lack of understanding and acknowledgement of BED as a psychological disorder, and allude to the misconception that BED is indistinguishable from obesity.

On a more societal level, personality attributions and perceptions of individuals with BED can offer further reasoning behind higher levels of stigma in relation to trivialness compared to AN and BN. Characteristics of laziness and carelessness are commonly associated with individual’s perceptions of BED [2]. Furthermore, perceptions around severity and control over the disorder also differentiate BED from AN and BN. Individuals with BED are seen to have more personal control over their condition, and BED itself is not recognised as being as serious a disorder as other EDs [19]. O’Connor et al. [19] found individuals to have particularly unfavourable views towards BED compared to AN and BN, perceiving BED as a lack of self-discipline rather than a psychological disorder. Such connotations demonstrate why public perceptions may lead to lesser consideration of BED as a debilitating psychological disorder in comparison to AN and BN and therefore attest to a differentiation in associated stigma, as demonstrated through higher levels of associated trivialness.

Additionally, research suggests that individuals are more likely to assume that a person with BED has a larger body [2, 18, 19, 36, 37]. Consequently, possible concurrent weight/obesity stigma may also contribute to higher levels of triviality stigma for BED, as higher levels of triviality could be due to individual’s perceptions of BED aligning more closely with obesity rather than a psychological disorder. In assessing the impact of weight stigma concurrent with BED, Hollett and Carter [18] found that individuals with both obesity and BED would face higher levels of cumulative stigma. Hollett and Carter [18] also found that only when eating behaviours of obese individuals are described (e.g., loss of control while eating), an increase in stigma becomes evident, regardless of whether a BED diagnosis exists. As such, the detected weight stigma associated with BED may result from pre-existing beliefs around obese individuals’ eating behaviours, rather than their body shape or weight per se. This interpretation further offers explanations towards the comparatively high levels of stigma in relation to triviality directed towards BED, as many people may consider loss of control while eating indicative of a lack of discipline or carless personality, rather than a symptom of psychopathology [18, 19].

### Limitations, implications, and future directions

Despite being one of the first empirical studies, to our knowledge, to measure the level of stigma attributed to AN, BN, and BED, in comparison with depression, our study was not without limitations. One of these is the use of convenience sampling, which no doubt led to selection bias. As a result of recruiting via university campuses and social media, our sample is likely not representative of the general population. This is evident in the high number of university-educated participants, thus making results less generalisable. Furthermore, due to recruiting across two university campuses, it is possible that participants largely came from particular undergraduate backgrounds, such as psychology and education. Consequently, participants with these backgrounds, or other tertiary education backgrounds, may reflect differing (and more likely lower) levels of stigma towards psychological disorders. Using more diverse recruitment strategies would help in limiting such an effect. Additionally, in providing participants with a description of the relevant disorder to their condition, participants’ responses may have been primed. For instance, highlighting certain characteristics such as extreme weight loss for AN or loss of control for BED could have influenced participants’ attitudes before completing the survey. This focus on disordered behaviours might have reinforced pre-existing stereotypes, such as perceiving individuals with AN as “disciplined” or those with BED as “weak” or “lacking self-control”. Future research could address this concern by not providing such descriptions, and therefore having participants respond without any educational tool to reflect pre-existing conceptions more accurately. Because participants responded to only one condition, it is possible that individuals with generally higher levels of stigma towards a specific psychological disorder were, by chance, assigned to that condition. Although random assignment aims to minimise this effect, having participants complete the scales for all conditions would allow for a more comprehensive investigation of stigma across the different disorders.

Another limitation was that a majority of our participants identified as being female, and our sample size was not adequate to study gender as a factor. Previous research has indicated that gender may influence ED stigma, both in the context of the individual with the ED and the individual holding stigmatising attitudes. O’Connor et al. [19] found attitudinal responses elicited by a vignette featuring a gender-neutral individual with BED was most likely to be judged as male, in comparison to counter vignettes featuring AN and BN. Wingfield et al. [20] also found that male participants thought ED recovery was easier to achieve than did females, while overall participants also identified a belief that it would be easier for a male to overcome an ED than a female. Evidently, embedded gendered stereotypes complicate the relationship between gender and ED stigma. It would be beneficial for future research to further disseminate the gender dynamic present in ED stigma to help tailor targeted destigmatisation interventions.

As research into ED stigma is still emerging, few scales have been developed which differentiate stigma associated with EDs specifically. As a result, the current study combined several existing scales. Although most scores on these scales demonstrated good internal consistency within the utilised sample, the adequacy of the scale’s sensitivity is unknown, thereby highlighting the possible difficulty in detecting more unique or subtle differences where they existed. Future research may benefit from using or developing an ED scale which specifically measures stigma associated with the different EDs. For example, it appears important to measure uncomfortableness due to emaciation associated with AN, or, as demonstrated in the current study, trivialness of diagnosis in BED.

Several practical implications can be identified from the current findings. With reference to both psychological disorders more broadly and EDs specifically, exacerbation of symptoms and reductions in help-seeking behaviour has been highly associated with stigma experiences [2, 19]. As a result, individuals with EDs may face fear in instigating conversations about suffering, and further may not be getting the support required for recovery. Given the devastating physiological effects seen within EDs, increases in symptom severity in combination with treatment delay or resistance can be fatal. This effect may be intensified when considering the trivialness associated with BED, as it appears the seriousness of BED as a psychological disorder is dismissed in comparison to AN or BN. Several previous public health campaigns demonstrate the effectiveness of national programs used to reduce stigma and discrimination in the context of psychological disorders and their impact on attitudes towards help seeking and treatment. Namely, an analysis of the effects of the *Time to Change* social marketing campaign in England demonstrated a reduction in levels of discrimination directed towards psychological disorders from several sources, including family, friends, neighbours, employers, and educational professionals [39].

Additionally, an evaluation of the effects of *Beyondblue: The National Depression Initiative* in Australia, suggested that the campaign had a positive effect on beliefs about depression treatment and the value of help-seeking behaviour, thereby suggesting the effectiveness of national awareness campaigns in improving community mental health knowledge [40]. Given the comparably lower levels of stigma currently associated with depression, the effects of such campaigns could be highly informative in efforts to lower stigma attributed to other psychological disorders, such as EDs.

Findings of the current study can also be used to inform intervention practices to help minimise the impact of stigma on individuals living with EDs. The well-established contact hypothesis [6] posits that negative attitudes result from lack of personal and positive contact. Brelet et al. [2] emphasised the applicableness of this social psychology hypothesis in the case of EDs in finding that being familiar with or having knowledge about EDs is associated with less attributed stigma. As such, in understanding the differences within community perceptions towards EDs and the different types of EDs, relevant organisations can aim to improve public awareness more specifically and further facilitate anti-stigma educational interventions as effective strategies to reduce ED stigma. Furthermore, knowledge regarding stigma towards specific types of EDs allows for tailored tolerance practices and training within ED treatment to help protect individuals with EDs from the harmful impacts consequential of stigma.

## Conclusions

In conclusion, our results may provide valuable insights into the stigmatisation of AN, BN, and BED, and demonstrate the wide array of negative attitudes and misunderstanding which surround EDs. It remains crucial that further research be conducted within this area to continue to provide knowledge and understanding around ED stigma, to ultimately reduce the burden on those affected by EDs.

## Data Availability

The datasets used and analysed during the current study are available from the corresponding author upon reasonable request.

## Footnotes

1 For the current study, the term “depression” will be used in reference to the diagnosis of major depressive disorder, as classified in the *DSM-5-TR* [11]. This is to avoid confusion surrounding specific diagnoses, as in the non-clinical population, major depressive disorder is commonly known and referred to as depression [25]. This also maintains consistency with previous research.

## Abbreviations

ED: Eating disorder
AN: Anorexia nervosa
BN: Bulimia nervosa
BED: Binge-eating disorder
SASS: Stigma and Self-Stigma Scale
OS: Opinions Scale
EDSS: Eating Disorder Stigma Scale
